# Prognostic Genes Linked to Asparagine Metabolism in Hepatocellular Carcinoma: Identification, Validation, and Regulatory Mechanisms Based on Transcriptome and Single-Cell RNA Sequencing

**DOI:** 10.3390/ijms27104425

**Published:** 2026-05-15

**Authors:** Jianting Feng, Kaihua Wei, Nana Li, Yinshi Li, Fei Du, Mengjiao Lv, Lifei Ma, Suwen Wang, Shuliang Niu, Liang Feng

**Affiliations:** 1 School of Basic Medical Sciences, Xinjiang Second Medical College, Karamay 834000, China; jianting2023@stu.shzu.edu.cn (J.F.); linana@xjsmc.edu.cn (N.L.); liyinshi@xjsmc.edu.cn (Y.L.); dufei@xjsmc.edu.cn (F.D.); lvmj@xjsmc.edu.cn (M.L.); malf@xjsmc.edu.cn (L.M.); nsliang@xjmu.edu.cn (S.N.); 2 College of Life Sciences, Shihezi University, Shihezi 832003, China; 3 School of Medicine, Shihezi University, Shihezi 832003, China; weikaihua@stu.shzu.edu.cn

**Keywords:** single-cell RNA, prognosis-related genes, hepatocellular carcinoma, asparagine metabolism

## Abstract

Metabolic reprogramming is closely linked to tumor proliferation, invasion, and immune escape. Despite its central role in amino acid metabolism, the regulatory mechanisms of asparagine metabolism in hepatocellular carcinoma (HCC) progression remain poorly characterized. Rather than focusing on canonical metabolic genes, prognostic markers were identified from co-expression modules associated with asparagine metabolism signatures. Using the TCGA database and asparagine metabolism-related gene sets, a prognostic risk-scoring model was developed through differential expression analysis, univariate Cox regression, and the LASSO algorithm and externally validated with the GEO dataset (GSE14620). Survival analysis, ROC curve evaluation, nomogram construction, scRNA-seq, GSEA, and drug sensitivity analysis were performed to systematically delineate the molecular mechanisms by which asparagine metabolism drives HCC progression. A three-gene signature comprising BOP1, SAC3D1, and PDE2A effectively stratified patients into high- and low-risk groups. High-risk patients exhibited markedly poorer overall survival, enrichment in tumor proliferation-associated pathways, increased tumor purity, reduced immune cell infiltration, and a substantially higher TP53 mutation rate (38% vs. 13%). In contrast, the low-risk group showed enrichment in pathways linked to hepatoblastoma suppression and liver function, alongside improved predicted response to immunotherapy. Single-cell analysis identified NK cells and endothelial cells as central mediators of asparagine metabolism-driven HCC progression, with BOP1, SAC3D1, and PDE2A displaying dynamic expression patterns during differentiation. Furthermore, the high-risk group was predicted to be more sensitive to chemotherapeutics such as cyclophosphamide and 5-fluorouracil. These findings highlight a potential interplay between nitrogen metabolism and asparagine metabolism in HCC and suggest mechanisms by which these pathways may influence NK cell and endothelial cell function to promote disease progression. This study establishes a novel prognostic model and identifies potential chemotherapeutic vulnerabilities in high-risk patients, warranting further experimental and clinical validation.

## 1. Introduction

Hepatocellular carcinoma (HCC) ranks among the top three causes of cancer-related mortality worldwide [1], representing 80–90% of primary liver cancer cases. Global cancer statistics for 2020 report approximately 906,000 new liver cancer cases and 830,000 deaths annually [2], reflecting a substantial disease burden. Key risk factors include chronic HBV and HCV infection, progression of alcoholic liver disease and NAFLD [3], and exposure to aflatoxin. Although targeted therapies such as sorafenib and Lenvatinib [4], along with immune checkpoint inhibitors including atezolizumab [5], have modestly improved outcomes for advanced HCC, the disease continues to face major clinical challenges, including late-stage diagnosis, postoperative 5-year recurrence rates exceeding 70%, and pronounced tumor drug resistance [6]. Consequently, identifying novel molecular targets is critical for developing precise therapeutic strategies.

Mechanistic studies indicate that HCC initiation and progression are closely linked to tumor metabolic reprogramming [7,8], particularly aberrant amino acid metabolism [9]. Dysregulated asparagine metabolism [10] influences prognosis by modulating the tumor microenvironment (TME) and facilitating immune escape [11,12], providing a rationale for targeted interventions based on metabolic pathways and gene regulation. Asparagine synthetase (ASNS), which catalyzes the conversion of aspartate to asparagine [13], is upregulated in HCC and promotes cell proliferation through activation of the mTOR signaling pathway [14]. Moreover, pituitary tumor-transforming gene 1 (PTTG1) enhances ASNS transcription by binding to its promoter [15], driving asparagine metabolic reprogramming and accelerating malignant progression [16]. Beyond directly regulating HCC cell proliferation and malignancy, aberrant asparagine metabolism also contributes to immune evasion by disrupting TME homeostasis. The reciprocal interactions between asparagine metabolism and the TME have therefore become a focal point in liver cancer research.

The TME constitutes a complex local ecosystem encompassing tumor cells [17], non-tumor cells, extracellular matrix (ECM), vasculature, immune cells, and additional components [18]. The establishment of an immunosuppressive microenvironment is closely associated with tumor cell-mediated immune escape. In HCC, intracellular asparagine levels within immune cells are markedly elevated [19]. Studies have demonstrated that inhibiting asparagine metabolism can suppress tumor growth by enhancing T-cell infiltration and promoting macrophage polarization toward a pro-inflammatory phenotype [20]. Targeted modulation of the asparagine metabolic pathway can therefore remodel the TME and augment antitumor immune responses, with inhibition of asparagine synthesis shown to decelerate tumor growth and improve immunotherapy efficacy.

Despite these findings, the precise molecular mechanisms through which asparagine metabolism-related biomarkers influence HCC initiation and progression remain unresolved. This study employed an integrated multi-omics approach to systematically investigate the role and regulatory mechanisms of asparagine metabolic signatures in HCC. By combining bulk transcriptomic data from the TCGA database with single-cell RNA sequencing, key genes associated with asparagine metabolism in HCC were identified. Machine learning algorithms were subsequently applied to develop a risk-scoring model for HCC patients, aiming to elucidate potential mechanisms by which these genes contribute to malignant progression through modulation of the tumor immune microenvironment. The findings offer novel insights and experimental evidence for refined prognostic assessment and targeted therapeutic strategies in HCC.

## 2. Results

### 2.1. Identification of Genes with Different Expression Levels in HCC and AMRG Score Expression Patterns in the Training Cohort

**Figure 1:**
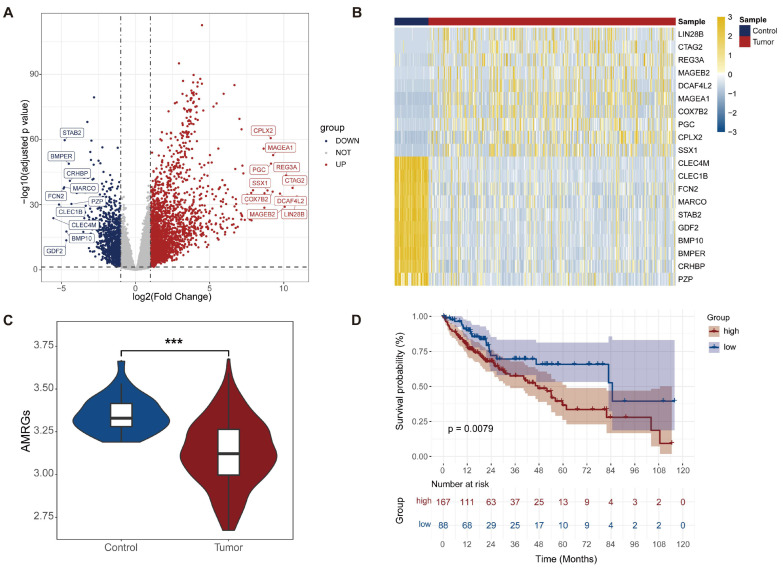
Analysis of differentially expressed genes and validation of prognostic stratification. (**A**) Volcano plot depicting DEGs between control and tumor groups; red and blue dots represent up- and downregulated genes, respectively, with the 10 most significantly altered genes labeled. (**B**) Heatmap illustrating expression levels of the top 10 up- and downregulated DEGs across samples; rows represent genes, columns represent samples. (**C**) Comparison of AMRG scores between paracancerous and HCC tissues. (**D**) Kaplan–Meier survival curves stratified by high and low AMRG score groups. *** *p* < 0.001.

Differentially expressed genes (DEGs) in HCC were identified using the DESeq2 package to compare gene expression between tumor tissues and adjacent non-tumor tissues from the TCGA-LIHC cohort. This analysis identified 3388 DEGs, including 2354 upregulated and 1034 downregulated genes. Among the upregulated genes, CPLX2, MAGEA1, PGC, REG3A, SSX1, CTAG2, COX7B2, DCAF4L2, MAGEB2, and LIN28B exhibited the most pronounced overexpression, whereas STAB2, BMPER, CRHBP, MARCO, FCN2, PZP, CLEC18, CLEC4M, BMP10, and GDF2 were the most strongly downregulated (Figure 1A). A heatmap of the top 10 up- and downregulated DEGs based on |log_2_FC| was generated using the ComplexHeatmap package to visualize expression patterns (Figure 1B), confirming consistent overexpression in HCC samples and underexpression in adjacent non-tumor tissues.

To assess the distribution of asparagine metabolism-related genes (AMRGs) across samples in the training cohort, ssGSEA was performed using the GSVA package to calculate enrichment scores. AMRG scores were significantly lower in adjacent non-tumor samples compared to HCC samples with complete survival data (*p* < 0.001) (Figure 1C). Kaplan–Meier survival analysis using the survival package demonstrated that overall survival declined more rapidly in the high-risk group than in the low-risk group, with HCC patients exhibiting high AMRG scores showing significantly poorer survival (*p* < 0.001) (Figure 1D).

Lower AMRG expression in tumors reflects metabolic remodeling during malignant transformation. Within HCC samples, a lower AMRG score indicates reduced reliance on asparagine-driven anabolic metabolism, correlating with a less aggressive phenotype and better prognosis, whereas higher AMRG activity is associated with enhanced proliferation and poorer survival.

### 2.2. Developing a Weighted Gene Co-Expression Network and Examining the Relationship Between Modules and AMRG Scores

**Figure 2:**
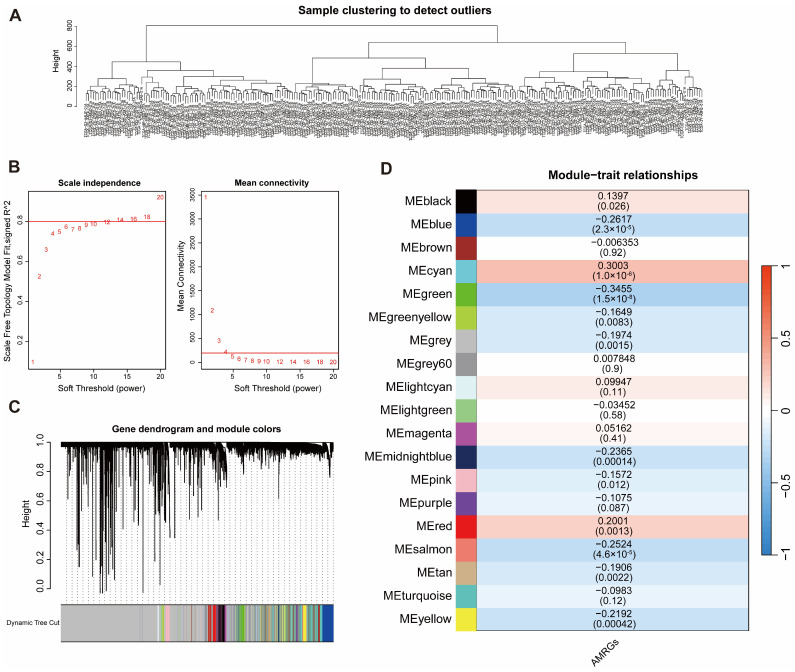
Weighted gene co-expression network (WGCNA) analysis. (**A**) Sample clustering dendrogram; (**B**) Scale-free topology fitting index and mean connectivity across soft-threshold powers; (**C**) Gene clustering dendrogram showing module assignment; (**D**) Heatmap of correlations between module eigengenes and AMRG scores.

Using the training set of HCC tissue samples with complete survival data, hierarchical clustering was performed with the hclust function to evaluate sample quality. No outliers were detected, indicating suitability for downstream analysis (Figure 2A). The optimal soft-thresholding power was determined using the pickSoftThreshold function, with a minimum β value of 16 achieving a scale-free topology fitting index (R^2^) above 0.8 (Figure 2B).

Gene module clustering was conducted via the WGCNA algorithm. Following topological overlap matrix transformation to reduce data noise, a minimum module size of 50 genes was applied for clustering and dynamic module identification, resulting in 19 distinct gene modules (Figure 2C). Module eigengenes (MEs) were calculated for each module, and their correlations with AMRG scores were evaluated. The MEcyan module (cor > 0.3, *p* < 0.05, 116 genes) exhibited a significant positive correlation, while the MEgreen module (cor < −0.3, *p* < 0.05, 500 genes) showed a significant negative correlation with AMRG scores, collectively yielding 616 core genes for further analysis (Figure 2D).

### 2.3. Functional Enrichment and Interaction Network Analysis of Differentially Expressed and Core Module Genes, and Construction of a Prognostic Model

**Figure 3:**
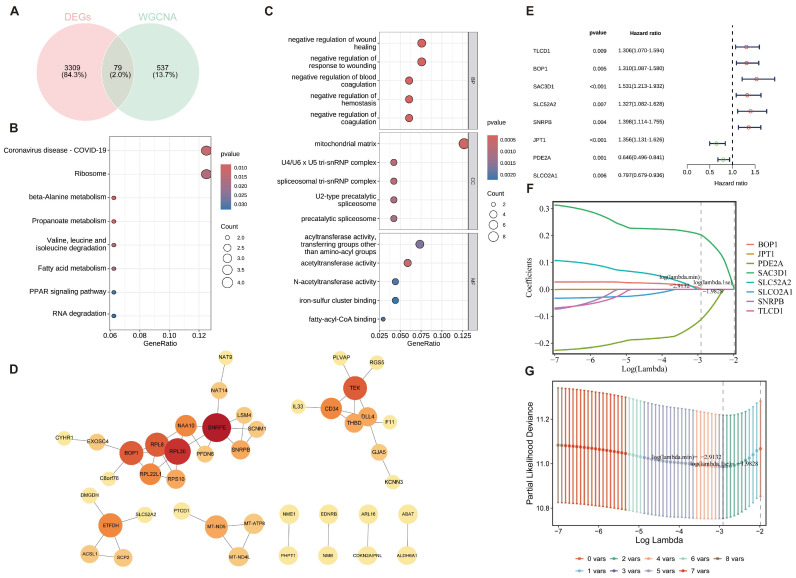
Screening of asparagine metabolism-related candidate genes and identification of prognostic genes in HCC. (**A**) Identification of candidate genes; (**B**) GO enrichment analysis; (**C**) KEGG enrichment analysis; (**D**) PPI network of candidate genes; (**E**) Univariate Cox regression analysis; (**F**) LASSO regression coefficient profile; (**G**) Cross-validation plot of the LASSO regression model.

Asparagine metabolism-related candidate genes were identified by intersecting 3388 DEGs with 616 module genes using the VennDiagram tool, yielding 79 overlapping candidates. Among these, 63 genes (80%) were upregulated and 16 (20%) were downregulated, with DEGs comprising 43% of the module genes and 13.7% of all DEGs (Figure 3A).

GO and KEGG enrichment analyses were performed using the clusterProfiler package in R to characterize the biological functions and signaling pathways of the candidate genes. A total of 319 GO terms (240 BPs, 44 CCs, 31 MFs) and 10 KEGG pathways were identified. KEGG analysis revealed significant enrichment in β-alanine metabolism (*p* = 2.1 × 10^−5^), propanoate metabolism (*p* = 5.1 × 10^−5^), and PPAR signaling pathways (*p* = 8.7 × 10^−5^) (Figure 3B), while GO enrichment highlighted involvement in RNA processing, metabolic processes, and related molecular functions (Figure 3C).

Protein–protein interaction (PPI) networks were constructed using the STRING database, resulting in a network of 43 nodes and 49 edges after removing isolated genes. The core genes RPL30 and SNRPF exhibited strong interaction connectivity (Figure 3D).

To identify genes with prognostic relevance, univariate Cox regression analysis was conducted using the survival package, revealing eight genes significantly associated with HCC patient survival: TIC1, SLC2A2, SNRPB, IATI, SLC2A1, BOP1, SAC3D1, and PDE2A. Hazard ratios (HR), 95% confidence intervals (CI), and *p*-values were visualized in a forest plot, confirming compliance with the proportional hazards assumption (Figure 3E). LASSO regression with ten-fold cross-validation was then applied to these eight genes using the glmnet package. The cross-validation curve identified the optimal λ for model fitting (Figure 3F), and the coefficient profile illustrated gene coefficient changes with λ (Figure 3G). Three genes with non-zero coefficients—BOP1, SAC3D1, and PDE2A—were ultimately selected as HCC prognostic genes.

### 2.4. Development and Validation of the Risk Prediction Model

**Figure 4:**
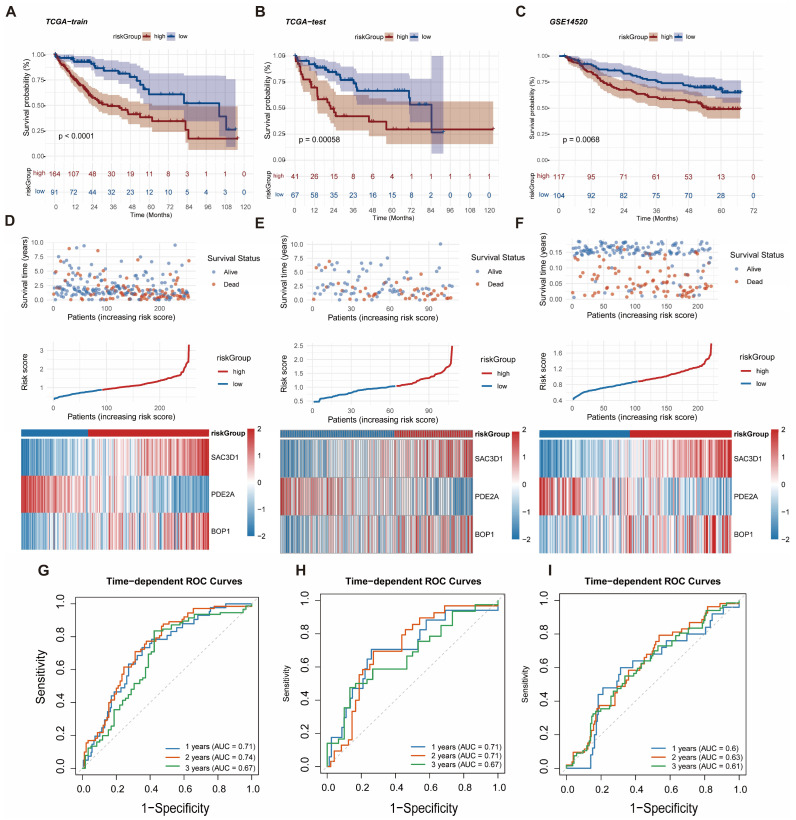
Survival and efficacy validation of the prognostic model across cohorts. (**A**–**C**) Kaplan–Meier survival curves for the TCGA training set, TCGA internal validation set, and GEO external validation set. (**D**–**F**) Risk score distributions, survival status distributions, and model gene expression heatmaps for the TCGA training set, TCGA internal validation set, and GEO external validation set. (**G**–**I**) 1-, 2-, and 3-year ROC curves for the TCGA training set, TCGA internal validation set, and GEO external validation set.

Kaplan–Meier survival curves were generated to compare high-risk and low-risk groups, and survival differences were assessed using log-rank tests across the TCGA training cohort, TCGA internal validation cohort, and GSE14620 external validation cohort. In the TCGA training cohort, the high-risk group exhibited a significantly lower survival probability than the low-risk group (*p* < 0.01; Figure 4A). This pattern persisted in the TCGA internal validation cohort, where high-risk patients demonstrated markedly reduced survival compared to low-risk patients (*p* = 0.0058; Figure 4B). Similarly, the GSE14620 external validation cohort confirmed significantly poorer survival outcomes for the high-risk group (log-rank test, *p* = 0.0068; Figure 4C). These findings indicate that the prognostic stratification provided by this risk model remains robust across independent datasets.

Three independent datasets were employed to validate the prognostic model’s performance. In the TCGA training cohort, analyses of risk score distributions, gene expression heatmaps, and survival status demonstrated a clear increase in risk scores with higher patient risk grades. BOP1, SAC3D1, and PDE2A expression levels were markedly elevated in parallel with rising risk scores, and the high-risk group exhibited a substantially greater proportion of deceased patients compared to the low-risk group (Figure 4D). Similar trends in risk score, gene expression, and survival status were observed in the TCGA internal validation cohort (Figure 4E). The GSE14620 external validation cohort further confirmed the model’s stability, showing increased risk scores with higher risk grades, elevated BOP1/SAC3D1/PDE2A expression in the high-risk group, and a significantly higher mortality rate among high-risk patients (Figure 4F).

Time-dependent ROC curves were used to evaluate the model’s predictive performance across all three cohorts. In the TCGA training cohort, AUC values for 1-, 2-, and 3-year survival were 0.71, 0.74, and 0.67, respectively (Figure 4G). The TCGA internal validation cohort showed AUCs of 0.71, 0.71, and 0.67 for 1-, 2-, and 3-year survival (Figure 4H). In the GSE14620 external validation cohort, 1- and 2-year survival AUCs were 0.63 and 0.61, respectively (Figure 4I). These results demonstrate that the prognostic model reliably predicts survival across multiple datasets, with particularly strong performance in the internal validation cohort.

### 2.5. Correlation Analysis of Clinicopathological Characteristics, Screening of Independent Prognostic Factors, and Construction and Validation of the Nomogram Model

**Figure 5:**
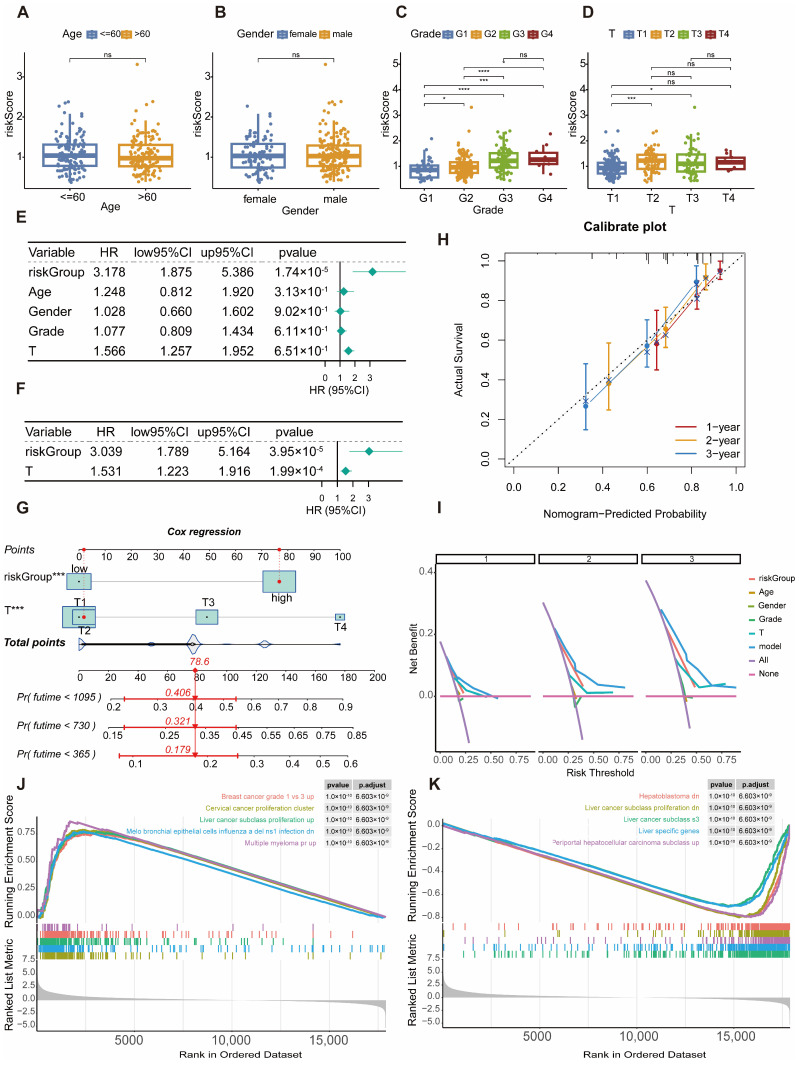
Correlation analysis of clinical characteristics and nomogram construction. (**A**) Risk score differences by age; (**B**) Risk score differences by gender; (**C**) Risk score differences by tumor grade; (**D**) Risk score differences by T stage; (**E**,**F**) Forest plots of univariate and multivariate Cox regression analyses; (**G**) Nomogram integrating risk score and independent prognostic factors to predict 1-, 2-, and 3-year survival; (**H**) Calibration curves; (**I**) Decision curves of the nomogram and clinical parameters; (**J**) GSEA enrichment analysis of the high-risk group; (**K**) GSEA enrichment analysis of the low-risk group. * *p* < 0.05; *** *p* < 0.001; **** *p* < 0.0001; ns, *p* > 0.05.

The relationship between risk stratification and clinicopathological features was examined using boxplots to visualize distribution patterns and the Kruskal–Wallis test to assess statistical significance. No significant differences in risk score distribution were observed between patients aged ≤60 years and >60 years (*p* > 0.05; Figure 5A), or between male and female patients (*p* > 0.05; Figure 5B). In contrast, risk scores varied significantly across tumor grade subgroups (G1–G4), with higher scores predominantly in high-grade tumors (G3, G4) and lower scores in low-grade tumors (G1, G2) (*p* < 0.05; Figure 5C). Similarly, T stage (T1–T4) was associated with significant differences in risk scores, with patients in T3 and T4 stages exhibiting higher scores than those in T1 and T2, reaching statistical significance specifically between T3 and T1 (*p* < 0.05; Figure 5D).

Univariate and multivariate Cox regression analyses identified independent prognostic factors. Univariate analysis revealed that riskGroup (HR = 3.178, 95% CI: 1.875–5.386, *p* = 1.74 × 10^−5^) and T stage (HR = 1.566, 95% CI: 1.257–1.952, *p* = 6.51 × 10^−5^) were significantly associated with overall survival, whereas age, gender, and tumor grade showed no significant correlation (Figure 5E). Multivariate analysis confirmed that both riskGroup (HR = 3.039, 95% CI: 1.789–5.150, *p* = 3.95 × 10^−5^) and T stage (HR = 1.531, 95% CI: 1.223–1.916, *p* = 1.99 × 10^−4^) functioned as independent prognostic factors (Figure 5F).

A nomogram integrating these two independent prognostic factors was developed (Figure 5G). Total scores, calculated by summing points assigned to riskGroup and T stage, enabled prediction of 1-, 2-, and 3-year survival probabilities. Calibration curves demonstrated strong concordance between nomogram-predicted and observed survival outcomes at all three time points (Figure 5H). Decision curve analysis indicated that the nomogram provided superior net clinical benefit compared to T stage or riskGroup alone, as well as relative to the “All” and “None” strategies across most threshold probabilities (Figure 5I).

To investigate biological mechanisms underlying differences between high- and low-risk groups, genome-wide GSEA was performed on the training cohort (samples with complete survival data) using the “DEseq2” package. High-risk patients exhibited enrichment of biological processes associated with HCC subtype proliferation, including pathways related to breast cancer grade and cervical cancer proliferation. Conversely, low-risk patients showed enrichment in hepatoblastoma-associated processes, downregulated HCC subtype proliferation, and liver-specific gene functions (Figure 5J,K).

### 2.6. Comparative Analysis of the Tumor Microenvironment and Immune Landscape Across Risk Groups

**Figure 6:**
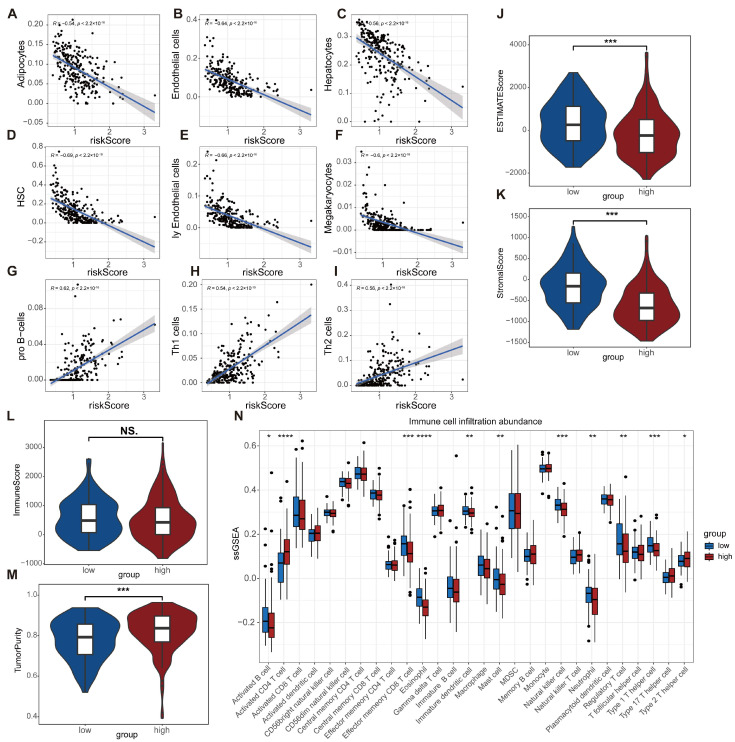
Correlation analysis of risk score and tumor immune microenvironment. (**A**–**I**) Scatter plots of correlations; (**J**–**M**) Violin plots of StromalScore, ImmuneScore, ESTIMATE score, and TumorPurity across risk groups; (**N**) Box plots of immune cell score differences. * *p* < 0.05; ** *p* < 0.01; *** *p* < 0.001; **** *p* < 0.0001; ns, *p* > 0.05.

The relationships among risk scores, the TME, and immune-related features were assessed, comparing high-risk and low-risk groups. Eosinophil and endothelial cell abundances exhibited significant negative correlations with risk score (r = −0.54, *p* < 0.001; r = −0.64, *p* < 0.001; Figure 6A–C), as did fibroblast and M2 macrophage infiltration levels (r = −0.69, *p* < 0.001; r = −0.66, *p* < 0.001; Figure 6D–F). Conversely, risk score correlated positively with inflammation-associated features, including pro-B cell and Th1 cell abundances (r = 0.62, *p* < 0.001; r = 0.54, *p* < 0.001; Figure 6G–I).

Comparative analysis revealed that the ES_MIKE score was significantly elevated in the high-risk group relative to the low-risk group (*p* < 0.05; Figure 6J). Additionally, StromalScore was higher in the high-risk group (*p* < 0.01; Figure 6K), whereas ImmuneScore did not differ significantly between groups (*p* > 0.05; Figure 6L). TumorPurity was also increased in the high-risk group compared to the low-risk group (*p* < 0.01; Figure 6M).

Analysis of immune cell infiltration patterns identified notable differences between groups, with the high-risk group displaying significantly reduced infiltration of activated B cells, effector memory CD8^+^ T cells, regulatory T cells, and Th1 cells (*p* < 0.05; Figure 6N).

### 2.7. Differential Analysis of Chemotherapeutic Drug Sensitivity, Immune Checkpoint Expression, TIDE Score, and Gene Mutation Characteristics Between High and Low Risk Groups

**Figure 7:**
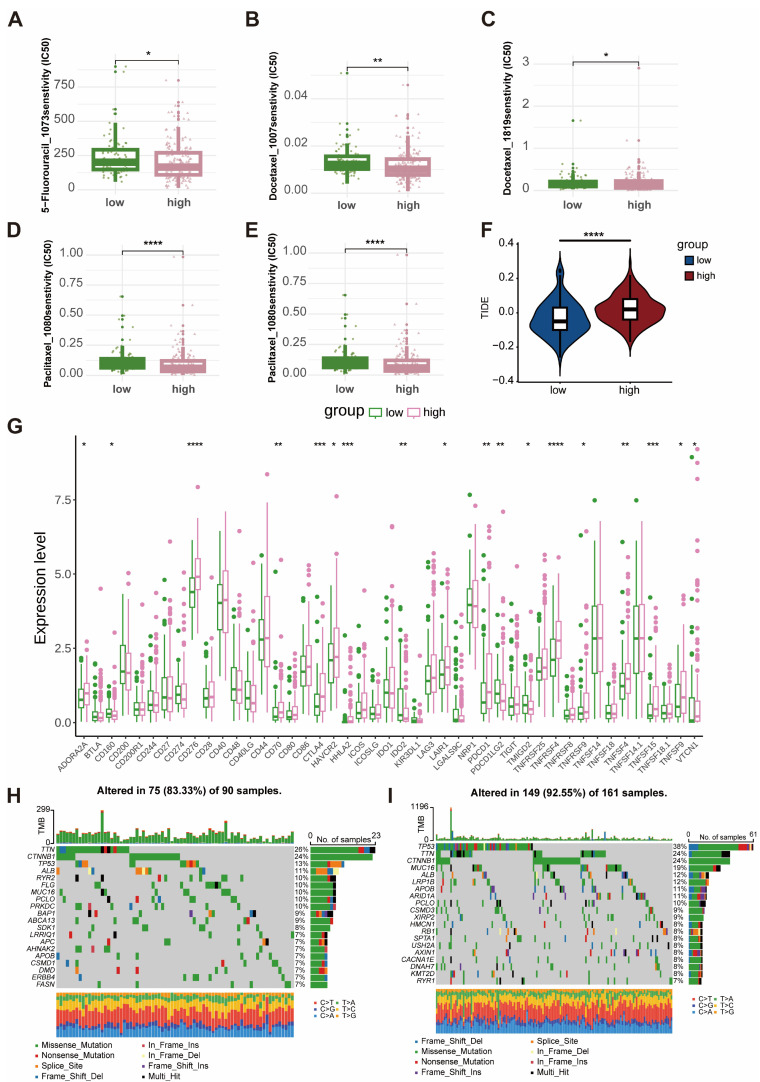
Differences in therapy-related characteristics and gene mutation profiles between high- and low-risk groups. (**A**) IC_50_ values of 5-fluorouracil; (**B**,**C**) IC_50_ values of docetaxel; (**D**) IC_50_ values of paclitaxel; (**E**) IC_50_ values of oxaliplatin; (**F**) TIDE score differences; (**G**) immune checkpoint gene expression; (**H**,**I**) mutation frequency plots of low- and high-risk groups. * *p* < 0.05; ** *p* < 0.01; *** *p* < 0.001; **** *p* < 0.0001.

Therapeutic characteristics of high-risk and low-risk groups in the training cohort were systematically compared across four dimensions: chemotherapeutic drug sensitivity, immune evasion traits, immune checkpoint expression, and gene mutation profiles. Predicted IC_50_ values for 5-fluorouracil, docetaxel, and oxaliplatin were significantly higher in the high-risk group (*p* < 0.05; Figure 7A–C).

TIDE analysis indicated a significantly elevated TIDE score in the high-risk group compared to the low-risk group (*p* < 0.001; Figure 7F). Among 45 conventional immune checkpoints, expression levels were markedly higher in the high-risk group (*p* < 0.001; Figure 7G).

Gene mutation profiling revealed mutations in 75 of 90 samples (83.3%) in the low-risk group (Figure 7H) and 149 of 161 samples (92.55%) in the high-risk group (Figure 7I), with mutation frequency notably higher in the high-risk cohort. Specifically, TP53 mutation rates were significantly elevated in the high-risk group (38%) relative to the low-risk group (13%) (*p* < 0.05). In contrast, mutation frequencies of CTNNB1 and TERT, other commonly mutated genes in HCC, did not differ significantly between groups, indicating that the prognostic model was particularly associated with TP53 inactivation rather than generalized oncogenic mutations.

### 2.8. Screening of Highly Variable Genes in Single-Cell Transcriptome and Analysis of Prognostic Gene Expression Characteristics

**Figure 8:**
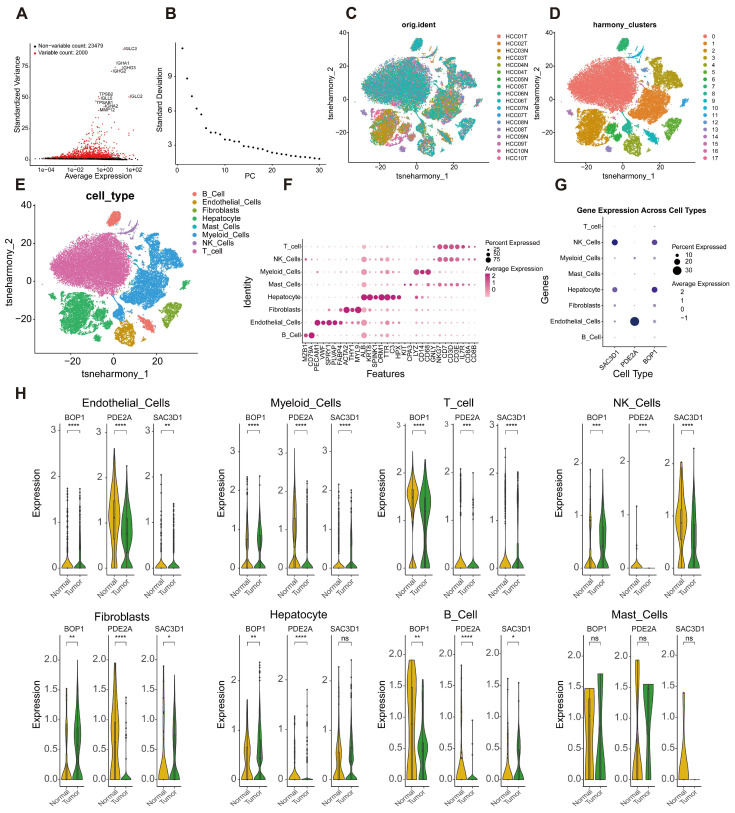
Cellular heterogeneity and prognostic gene expression in HCC single-cell transcriptome data. (**A**) Identification of highly variable genes; (**B**) Scree plot; (**C**) PCA clustering dimensionality reduction; (**D**) tSNE clustering dimensionality reduction; (**E**) Distribution of cell types; (**F**) Bubble plot of differentially expressed genes; (**G**) Bubble plot of key prognostic genes across cell types; (**H**) Prognostic gene expression plot. * *p* < 0.05; ** *p* < 0.01; *** *p* < 0.001; **** *p* < 0.0001; ns, *p* > 0.05.

To characterize cellular heterogeneity in HCC and the expression patterns of prognosis-associated genes, a single-cell transcriptome dataset was analyzed. Two thousand highly variable genes exhibiting significant differential expression across distinct cell clusters were identified and selected as core genes for downstream analyses (Figure 8A). Principal component analysis (PCA) of these genes indicated that the first 20 principal components accounted for the majority of variance in the dataset (Figure 8B). Dimensionality reduction and clustering subsequently partitioned all cells into 17 distinct clusters, and the distribution of sample sources across these clusters was examined to define the cellular composition of each sample (Figure 8C,D).

Cell-type annotation was performed by integrating literature-derived marker genes with the SingleR tool, classifying the 17 clusters into 8 major cell types: fibroblasts, B cells, T cells, NK cells, myeloid cells, endothelial cells, mast cells, and hepatocytes, thereby resolving the cellular composition of the dataset (Figure 8E). A bubble plot visualized characteristic marker gene expression and the proportion of cells expressing each marker (Figure 8F).

Expression profiles of the three prognostic genes used in the prognostic model were further examined. Violin plot analysis demonstrated that these genes were predominantly and significantly overexpressed in NK cells and endothelial cells within the HCC cohort (*p* < 0.05), identifying these two cell types as key subsets for subsequent HCC studies (Figure 8H).

### 2.9. Analysis of Single-Cell Trajectory Evolution, Cellular Interactions, and Functional Characteristics in HCC

**Figure 9:**
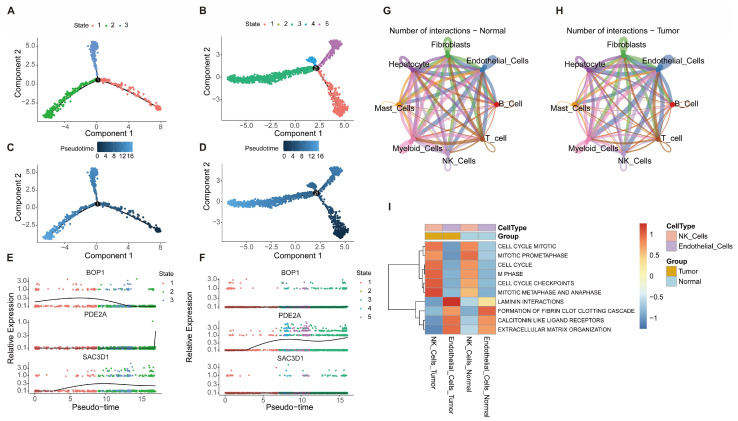
Analysis of single-cell trajectory evolution, cellular interaction networks, and functional characteristics in HCC. (**A**,**B**) Pseudotime trajectory plots of HCC cells; (**C**,**D**) Pseudotime trajectory plots of paracancerous normal cells, compared to tumor cells; (**E**,**F**) Dynamic expression profiles of core prognostic genes during pseudotime evolution in tumor cells (**E**) and normal cells (**F**); (**G**,**H**) Cellular interaction networks in normal tissues (**G**) and HCC tissues (**H**); (**I**) Functional enrichment heatmap of cells in HCC and normal groups, illustrating differences in pathway enrichment scores. Malignant hepatocytes in this study were isolated from HCC tumor tissues and distinguished from normal hepatocytes derived from non-tumor tissues based on specific marker genes and transcriptomic features.

To elucidate the dynamic evolution of HCC cells and the interactive characteristics of the TME, single-cell trajectory analysis, cellular interaction network analysis, and functional enrichment analysis were conducted. Pseudotime analysis revealed that NK cells progressed through three continuous states, while endothelial cells transitioned through five states, reflecting gradual progression from an initial to a malignant phenotype (Figure 9A,B). Distinct differences were observed between the evolutionary trajectories of NK cells and endothelial cells (Figure 9C,D). Expression levels of the core prognostic genes BOP1, PDE2A, and SAC3D1 increased progressively along tumor cell evolutionary states (Figure 9E,F).

Cellular interaction network analysis indicated that hepatocytes, fibroblasts, endothelial cells, and other cell types in HCC tissues exhibited a higher number of interactions and stronger interaction intensity compared to normal tissues (Figure 9G,H). Functional enrichment analysis demonstrated that cell cycle-related processes, including cell cycle mitotic, mitotic prometaphase, cell cycle, M phase, cell cycle checkpoints, and mitotic metaphase and anaphase, as well as pathways such as laminin interactions, formation of fibrin clot clotting cascade, calcitonin-like ligand receptors, and extracellular matrix organization, displayed differential enrichment or activity among cell types and between normal and tumor groups (Figure 9I).

Quantitative analyses confirmed NK cells in three pseudotime states and endothelial cells in five states. Cell–cell communication analysis revealed significantly enhanced interaction strength and ligand–receptor pair engagement among hepatocytes, endothelial cells, and NK cells in HCC tissues compared to normal tissues. Functional enrichment highlighted that these two cell types are highly involved in cell cycle regulation, immune activity, and ECM organization, supporting their roles as core populations in asparagine metabolism-mediated HCC progression.

## 3. Discussion

Due to the absence of specific clinical symptoms, HCC is often diagnosed at an advanced stage, with many patients missing the optimal window for surgical intervention. Despite advances in cancer prevention [21], early screening, and recent therapeutic strategies, HCC prognosis remains poor, and survival rates continue to be low [22], highlighting the urgent need to identify novel therapeutic targets to improve treatment outcomes. Metabolic dysregulation is a central feature of HCC, yet its precise regulatory mechanisms remain incompletely understood.

BOP1, SAC3D1, and PDE2A were identified as prognostic genes from co-expression modules associated with asparagine metabolism-related transcriptional patterns, although they do not act as direct metabolic enzymes. While the roles of core metabolic pathways, such as glycolysis and lipid metabolism, in tumor progression [23] and immune evasion [24] are well documented, the mechanisms driving imbalanced amino acid metabolism remain poorly characterized. Metabolomic analyses of serum from liver cancer patients have identified aspartate, glutamate, and taurine as the most significantly altered metabolites during HCC development [25]. Dysregulated amino acid uptake is recognized as a hallmark of cancer metabolism [26], suggesting that disturbed amino acid homeostasis plays a key regulatory role in HCC progression [27]. This study focused on AMRGs to systematically identify key molecules and regulatory networks associated with poor prognosis in HCC. Although a direct regulatory link between BOP1, SAC3D1, and PDE2A and ASNS has not been fully established, these genes are significantly associated with asparagine metabolism pathways. They may indirectly influence ASNS transcription, aspartate-to-asparagine conversion, and downstream mTOR signaling by modulating ribosome biogenesis, RNA transport, and cAMP/cGMP homeostasis, thereby promoting malignant progression. Using an integrated multi-omics approach, a novel three-gene prognostic signature was constructed, enabling high-precision risk stratification of HCC patients while revealing a distinct molecular mechanism by which HCC cells exploit asparagine metabolic reprogramming to establish an immunosuppressive microenvironment and drive tumor malignancy.

The central finding of this study is the identification of a core prognostic signature comprising BOP1, SAC3D1, and PDE2A, which collectively contribute to HCC cell proliferation, survival, and malignant phenotype formation under metabolic stress. BOP1 facilitates ribosome assembly, supporting sustained proliferation and resistance to apoptosis. SAC3D1 enhances RNA stability and transport efficiency, improving cellular adaptation to metabolic stress. PDE2A modulates cAMP/cGMP and mTOR signaling and is closely linked to asparagine-driven anabolic metabolism and tumor progression. BOP1 is markedly overexpressed in HCC tissues and, as a key regulator of ribosome biogenesis, its elevated expression promotes protein synthesis in tumor cells [28], providing resources for rapid proliferation while maintaining ribosomal homeostasis to tolerate metabolic stress. SAC3D1 influences transcriptional and translational efficiency of oncogenes via RNA transport and metabolic regulation [29], facilitating tumor invasion, metastasis, and clonal evolution. PDE2A regulates downstream proliferative and anti-apoptotic signaling [30] by balancing intracellular cAMP/cGMP pathways, and its dysregulated overexpression enhances metabolic adaptability and therapeutic resistance [31]. The concurrent dysregulation of these three genes forms the molecular basis for asparagine metabolic disturbance, aberrant cell cycle activation, and heightened malignancy in HCC [32]. Further analysis demonstrates that this signature is closely linked to the asparagine production pathway mediated by ASNS, suggesting a cooperative role in promoting tumor cell survival and proliferation under nutrient deprivation and metabolic stress through regulation of amino acid supply, mTOR activation, and redox homeostasis.

Beyond intrinsic metabolic adaptations, this study reveals that aberrant expression of AMRGs actively shapes an immunosuppressive TME. Immune infiltration analysis indicates that high-risk patients exhibit reduced infiltration of anti-tumor immune cells, including activated B cells, effector memory CD8^+^ T cells, regulatory T cells, and Th1 cells. Conversely, M2-type macrophages and fibroblast abundances are inversely correlated with the risk score. High-risk patients also show elevated immune checkpoint expression and higher TIDE scores, reflecting pronounced immune escape. Prior studies have shown that dysregulated amino acid metabolism in the TME [33] directly suppresses tumor-infiltrating lymphocyte activation and cytotoxic function [34], promoting T-cell exhaustion and dysfunction. This study further demonstrates that high expression of BOP1, SAC3D1, and PDE2A is significantly associated with these immunosuppressive features, indicating that asparagine metabolic reprogramming not only confers a proliferative and invasive advantage to HCC cells but also facilitates immune evasion by remodeling the TME and impairing immune cell function.

The markedly higher TP53 mutation rate in the high-risk group, coupled with comparable mutation frequencies of CTNNB1 and TERT between groups, highlights a specific association between the prognostic signature and TP53-driven malignant progression in HCC.

Single-cell transcriptomic analysis offers a high-resolution perspective, further elucidating the cellular distribution of asparagine metabolism-related prognostic genes and their regulatory roles within the TME. NK cells and endothelial cells were identified as key functional populations driving metabolic and malignant progression [35,36]. NK cells modulate antitumor immune surveillance, and asparagine metabolism influences their metabolic fitness and cytotoxic capacity; elevated metabolic activity in the TME impairs NK cell infiltration and function, promoting immune suppression and tumor progression [37]. Endothelial cells mediate angiogenesis and metabolic crosstalk, with asparagine metabolism supporting their proliferation and angiogenic activity, thereby accelerating tumor growth and invasion.

BOP1, SAC3D1, and PDE2A exhibit dynamic expression within these cell subsets, with significant upregulation during malignant progression. BOP1 sustains ribosome biogenesis under metabolic stress, maintaining protein synthesis and supporting rapid tumor cell proliferation. SAC3D1 ensures efficient RNA processing and metabolic homeostasis, enhancing tumor cell adaptability and survival under stress. PDE2A modulates intracellular cAMP/cGMP balance and downstream mTOR signaling, functionally integrated with asparagine-mediated anabolic metabolism to drive malignant proliferation. These processes reinforce aggressive tumor phenotypes and poor prognosis in high-risk patients.

These finely tuned regulatory dynamics at the levels of cellular heterogeneity and cell–cell interactions are difficult to capture with conventional bulk transcriptomic analyses and provide critical experimental evidence for dissecting the molecular mechanisms by which metabolic reprogramming mediates immunosuppression in HCC.

From a translational medicine perspective, this study highlights the clinical therapeutic potential of targeting key asparagine metabolism genes. The prognostic model based on BOP1, SAC3D1, and PDE2A demonstrates robust and efficient risk stratification across multiple TCGA and GEO cohorts, with 1- to 3-year predictive AUC values outperforming traditional clinicopathological indicators. This model provides a reliable tool for early risk assessment and individualized prognostic management of HCC. Integration with a nomogram further enhances clinical applicability, enabling rapid identification of high-risk patients and guiding targeted, stratified interventions. Regarding therapeutic development, the three core genes present actionable targets: BOP1 inhibition impedes ribosome synthesis and tumor proliferation; SAC3D1 targeting attenuates RNA metabolism-driven invasion and metastasis; and PDE2A intervention disrupts intracellular second-messenger homeostasis, promoting tumor cell apoptosis. These strategies offer a foundation for metabolism-targeted combination therapies in HCC patients with immunotherapy resistance.

Despite these advances, certain limitations remain. The study relies primarily on retrospective analyses of public datasets. Although multi-cohort validation reinforces result robustness, experimental verification in cellular and animal models is necessary. Functional validation experiments using animal models and clinical samples are currently underway to address this gap.

In conclusion, this study systematically demonstrates the pivotal role of asparagine metabolic reprogramming in HCC progression and immune evasion and identifies a three-gene prognostic signature centered on BOP1, SAC3D1, and PDE2A. It establishes a mechanistic link between amino acid metabolic disturbance and immunosuppressive TME formation, provides a high-accuracy prognostic stratification tool, and identifies potential therapeutic targets. These findings offer a novel framework for understanding metabolic-immune regulation in liver cancer, support the development of metabolism-targeted combination immunotherapy strategies, and open new avenues for overcoming current therapeutic limitations in HCC.

## 4. Materials and Methods

### 4.1. Data Collection

TCGA-LIHC transcriptome data were obtained from the TCGA database, including histologically confirmed HCC samples with matched tissue, sample identifiers labeled “01A” at positions 14–16, and complete survival information. A total of 363 tumor samples and 50 adjacent non-tumor samples were included and randomly divided into training and internal validation cohorts at a 7:3 ratio. The external validation cohort GSE14620 (GEO, GPL3921) comprised 221 HCC samples with complete survival data. Additionally, the single-cell RNA-seq dataset GSE146276 (GEO) contained 10 HCC and 8 normal liver samples. AMRGs were extracted from GeneCards (https://www.genecards.org/, accessed on 13 September 2025) using the terms “asparagine metabolism,” “asparagine synthesis,” “ASNS,” and “asparagine synthetase,” with genes scoring > 3 selected for subsequent analyses.

### 4.2. Identification of Differentially Expressed Genes

Differential expression analysis between HCC and adjacent non-tumor samples (TCGA-LIHC) was performed using the R package “DESeq2” (v1.40.2) with thresholds of adjusted *p*-value < 0.05 and |log_2_FC| > 1. Volcano plots were generated via “ggplot2” (v3.4.1), and “ComplexHeatmap” visualized expression patterns of the top 10 upregulated and downregulated DEGs.

### 4.3. Weighted Gene Co-Expression Network Analysis

AMRG scores for each sample were calculated using ssGSEA via the R package “GSVA” (v1.42.0). A weighted gene co-expression network was constructed with “WGCNA” (v1.73): hierarchical clustering of samples was performed using hclust, the optimal soft-thresholding power (β = 16) was determined by pickSoftThreshold to achieve scale-free topology (fitting index > 0.80), and genes were clustered into modules (minimum size = 50) based on the topological overlap matrix. Modules significantly correlated with the AMRG score (|cor| > 0.3, *p* < 0.05) were selected for further analysis.

### 4.4. Screening of Candidate Genes and Functional Enrichment Analysis

Candidate genes were defined as the intersection of DEGs and AMRG-related module genes using the R package “VennDiagram” (v1.7.1). GO and KEGG enrichment analyses were conducted with “clusterProfiler” (v4.2.2, *p* < 0.05), and ggplot2 visualized the top five GO terms and eight KEGG pathways. Candidate genes were uploaded to STRING (https://string-db.org, accessed on 13 September 2025), and a PPI network was constructed in Cytoscape (v3.9.1).

### 4.5. Construction and Validation of the Prognostic Model

In the training cohort, univariate Cox regression (R package “survival,” v3.7.0) identified genes with *p* < 0.01 and HR > 1, with proportional hazards assumptions verified via cox.zph (*p* > 0.05). LASSO regression with 10-fold cross-validation (“glmnet” v4.1.8) determined the optimal λ, selecting prognostic genes with non-zero coefficients. A multivariate Cox regression model generated the risk score: Risk score = 0.2492 × SAC3D1 − 0.23278 × PDE2A + 0.07989 × BOP1. Patients were stratified into high- and low-risk groups based on the optimal cutoff from the risk score distribution.

Kaplan–Meier survival curves were plotted using “survminer” (v0.4.9) and compared by log-rank test. Time-dependent ROC curves at 1-, 2-, and 3-year intervals were generated with “timeROC” (v0.4) to calculate AUC values, visualized via ggplot2. Risk score distributions, survival status, and module gene expression were displayed using “pheatmap” (v1.0.12).

### 4.6. Analysis of Risk Score and Clinical Characteristics

Subgroup analyses in the training cohort stratified patients by age (≤60 vs. >60 years), gender, tumor grade (G1–G4), and T stage (T1–T4). Associations between risk scores and clinical characteristics were assessed using the Wilcoxon rank-sum test for binary variables and the Kruskal–Wallis test for multi-category variables.

### 4.7. Independent Prognostic Analysis and Nomogram Construction

Univariate and multivariate Cox regression analyses (R package “survival”) identified independent prognostic factors. Variables with HR > 1 and *p* < 0.05 in univariate analysis were included in multivariate modeling, with proportional hazards assumptions verified (*p* > 0.05). A nomogram predicting 1-, 2-, and 3-year OS was constructed using the “rms” package, and its calibration and clinical utility were evaluated via calibration curves (“regplot”) and decision curve analysis (“ggDCA”).

### 4.8. Gene Set Enrichment Analysis (GSEA)

Gene set variation analysis (DESeq2) compared high- and low-risk groups in the training cohort. Genes were ranked by |log_2_FC|, and GSEA was performed using “c2.cp.kegg.v7.2.symbols.gmt” (MSigDB) via “clusterProfiler” with thresholds NES > 1, FDR < 0.25, and *p* < 0.05. The top ten enriched pathways were visualized.

### 4.9. Immune Infiltration Analysis

Infiltration levels of 64 immune cell types were quantified using xCellAnalysis (R package “xCell, version 1.3-8”), and Pearson correlation analysis (|cor| > 0.5, *p* < 0.05) assessed associations between risk scores and immune infiltration. Stromal score, immune score, ESTIMATE score, and tumor purity were calculated with the “estimate” package and compared between risk groups using the Wilcoxon rank-sum test. Infiltration of 28 immune cell types was additionally evaluated via ssGSEA.

### 4.10. Analysis of Drug Sensitivity and Immunotherapy Response

HCC chemotherapeutic sensitivity data were obtained from CDSC, and IC_50_ values were computed using the pRRophetic function (“oncoPredict”), with comparisons between high- and low-risk groups performed via Wilcoxon rank-sum test. Expression levels of 45 immune checkpoints were analyzed using the same test and visualized as boxplots. TIDE scores (official website) were used to evaluate differences in predicted immunotherapy response between groups.

### 4.11. Mutation Analysis

Somatic mutation profiles in the training cohort were analyzed using the oncoplot function from R package “maftools” to compare high- and low-risk groups.

### 4.12. Single-Cell RNA Sequencing Analysis

Single-cell RNA-seq data (GSE149614) were processed in Seurat (v5.0.1) with quality control thresholds: nFeature_RNA > 200, nCount_RNA > 2500, and percent.mt < 10%. After normalization, the top 2000 highly variable genes (vst method) were subjected to PCA and t-SNE clustering (resolution = 0.3). Cell clusters were annotated into eight major cell types using canonical literature markers and SingleR (v2.4.0). DotPlot visualized prognostic gene expression, and Wilcoxon rank-sum tests identified cell types with significant expression differences between HCC and normal samples. Key cell type analyses included pseudotime trajectory inference (“monocle” v1.6.1), intercellular communication assessment (“CellChat” v1.3.2), Reactome pathway enrichment (“GSVA” v1.42.0, FDR < 0.05), and differential pathway visualization using “pheatmap.” Cell type annotations employed canonical markers: hepatocytes (ALB), endothelial cells (PECAM1, CDH5), NK cells (NKG7, GNLY), T cells (CD3D, CD3E), B cells (CD19, MS4A1), myeloid cells (CD68, CD14), fibroblasts (COL1A1, DCN), and mast cells (TPSAB1, CPA3), with annotation reliability validated against reference datasets using SingleR.

### 4.13. Statistical Analysis

All statistical analyses were performed in R (v4.2.2). Comparisons between two groups used the Wilcoxon rank-sum test or t-test, while multi-group comparisons employed the Kruskal–Wallis test. Kaplan–Meier survival analysis was conducted with the log-rank test, and Pearson correlation analysis evaluated associations. Significance levels were indicated as follows: ****: *p* < 0.0001, ***: *p* < 0.001, **: *p* < 0.01, *: *p* < 0.05, and ns: *p* > 0.05.

## 5. Conclusions

This study systematically elucidated the role of AMRGs in the initiation and progression of HCC through multidimensional bioinformatics analyses. The constructed three-gene prognostic model demonstrated favorable predictive performance and showed strong associations with the TME, clinical characteristics, and treatment sensitivity. This study identified novel candidate prognostic biomarkers for HCC and suggests a potential theoretical direction for therapeutic strategies targeting asparagine metabolism, although further experimental validation is required. Limitations remain due to the complexity of biological systems. Future work will integrate experimental validation with multi-omics analyses to further elucidate regulatory mechanisms of AMRGs and facilitate clinical translation of these findings.

## Data Availability

The datasets (GSE149614) generated and/or analysed during the current study are available in the [GEO] repository, [https://www.ncbi.nlm.nih.gov/geo/, accessed on 13 September 2025].
